# Electrothermal Actuators for SiO_2_ Photonic MEMS

**DOI:** 10.3390/mi7110200

**Published:** 2016-11-07

**Authors:** Tjitte-Jelte Peters, Marcel Tichem

**Affiliations:** Precision and Microsystems Engineering (PME), Delft University of Technology, Mekelweg 2, 2628 CD Delft, The Netherlands; t.j.peters@tudelft.nl

**Keywords:** bimorph actuator, photonic waveguide, silicon reinforcement, curvature, deflection, bending stiffness, transient response

## Abstract

This paper describes the design, fabrication and characterization of electrothermal bimorph actuators consisting of polysilicon on top of thick (>10 μm) silicon dioxide beams. This material platform enables the integration of actuators with photonic waveguides, producing mechanically-flexible photonic waveguide structures that are positionable. These structures are explored as part of a novel concept for highly automated, sub-micrometer precision chip-to-chip alignment. In order to prevent residual stress-induced fracturing that is associated with the release of thick oxide structures from a silicon substrate, a special reinforcement method is applied to create suspended silicon dioxide beam structures. The characterization includes measurements of the post-release deformation (i.e., without actuation), as well as the deflection resulting from quasi-static and dynamic actuation. The post-release deformation reveals a curvature, resulting in the free ends of 800 μm long silicon dioxide beams with 5 μm-thick polysilicon to be situated approximately 80 μm above the chip surface. Bimorph actuators that are 800 μm in length produce an out-of-plane deflection of approximately 11 μm at 60 mW dissipated power, corresponding to an estimated 240 ${}^{\circ }C$ actuator temperature. The delivered actuation force of the 800 μm-long bimorph actuators having 5 μm-thick polysilicon is calculated to be approximately 750 μN at 120 mW.

## 1. Introduction

Combining suspended photonic waveguides and microactuators enables positionable waveguides, resulting in the photonic switch [1,2,3] and numerous other Micro-Opto-Electro-Mechanical Systems (MOEMS) applications [4,5,6]. However, the majority of research on positionable waveguides is limited to silicon photonic devices. The positionable waveguides presented in this paper are based on silicon nitride (Si${}_{3}$N${}_{4}$) waveguide cores within a thick (∼16 μm) SiO${}_{2}$ cladding. We propose the name ‘SiO${}_{2}$ photonic MEMS’ for this platform, due to the mechanically-dominant material being oxide.

**Figure 1:**
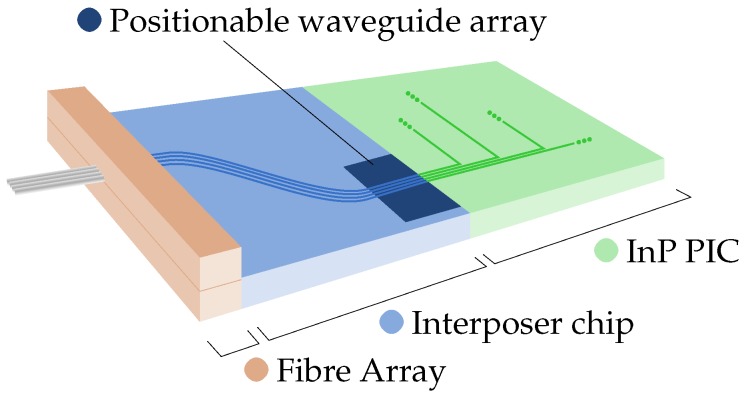
Alignment assembly with the fiber array, interposer chip and indium phosphide (InP) photonic integrated circuit (PIC). The location of the positionable waveguide array on the interposer chip is indicated.

We have explored the use of suspended SiO${}_{2}$ waveguides with integrated actuators as part of a novel chip-to-chip alignment concept for photonic integrated circuits (PICs). This concept is based on a two-stage assembly process, in which two PICs are first coarsely aligned and locked in position, after which the waveguides of the PICs are fine-aligned and locked in position. The strength of the alignment approach lies in a positionable waveguide array that is locally realized in one of the PICs and which is equipped with on-chip MEMS actuators to achieve sub-micrometer precise alignment with the waveguides of the other PIC. Figure 1 shows an illustrative case in which a fiber array is coupled to an indium phosphide (InP) PIC through an interposer chip with a positionable waveguide array. We emphasize that this is only one of many possible applications using positionable waveguides, and in this paper, we focus on the characteristics of the bimorph actuators in general.

The interposer chip, on which the positionable waveguide array is realized, is based on the TriPleX photonic platform [7], comprising ∼200 nm-thin silicon nitride (Si${}_{3}$N${}_{4}$) cores embedded in a ∼16 μm-thick SiO${}_{2}$ cladding. This photonic material stack, which is realized on top of a silicon substrate, poses challenges when used as a separate mechanical material.

The release of thick (>10 μm) SiO${}_{2}$ structures from their silicon substrate is afflicted by buckling of the structures, due to compressive stress in the SiO${}_{2}$. The buckled structures generate stress concentrations, which can lead to fracturing. Reports on suspended SiO${}_{2}$ structures with a thickness around 15 μm (the total thickness required for the TriPleX platform), a width of ∼18 μm and a length of roughly 1000 μm are not abundant in the literature. Ollier [2] reported on the realization of suspended structures of 25 μm-thick PECVD SiO${}_{2}$ using isotropic reactive ion etching (RIE). The problem of mechanical structures breaking is mentioned, as well as a solution: anti-buckling structures in combination with a specific Si etching process. Cooper et al. [8] demonstrated suspended silica waveguides with a cross-section of 100 μm × 40 μm. Their waveguide material is thermal SiO${}_{2}$ in combination with silica created by flame hydrolysis deposition (FHD), and the suspended waveguides are realized by a combination of dicing and wet etching.

The current work presents the design, fabrication and characterization of suspended TriPleX structures that are positionable by integrated out-of-plane actuators. Thermal bimorph actuators are realized by poly-Si heaters that are deposited on top of SiO${}_{2}$ beams. In earlier work, we reported on the initial fabrication results of thermal SiO${}_{2}$ suspended structures without photonic waveguide cores [9] and suspended TriPleX structures with waveguide cores [10]. In addition, we demonstrated TriPleX structures with waveguide cores and simple actuator designs [11], based on a backside etch approach to release the structures without silicon reinforcement. In the current work, the release of structures relies on front-side etching and benefits from a special silicon reinforcement method, greatly increasing the yield of suspended structures. Moreover, the bimorph actuators use the poly-Si as the heater material, obviating the need for additional metal heaters.

This paper first discusses the design of the structures and the actuators (Section 2), followed by the fabrication process details (Section 3). Section 4 includes the characterization of the post-release deformation and the actuator stroke. In Section 5, the actuator performance is discussed in light of the photonic alignment solution, and the conclusions are summarized.

## 2. Design

The requirements imposed on the positionable waveguide arrays follow from the misalignment after the first assembly stage. Flip chip bonding, one of the available pre-alignment methods for the first stage, aims to place the top surfaces of the interposer chip and the PIC in the same horizontal plane. The tolerance that can be achieved with flip chip bonding is in the order of one or two micrometers in out-of-plane alignment and several micrometers in in-plane alignment.

### 2.1. Out-of-Plane Actuators

**Figure 2:**
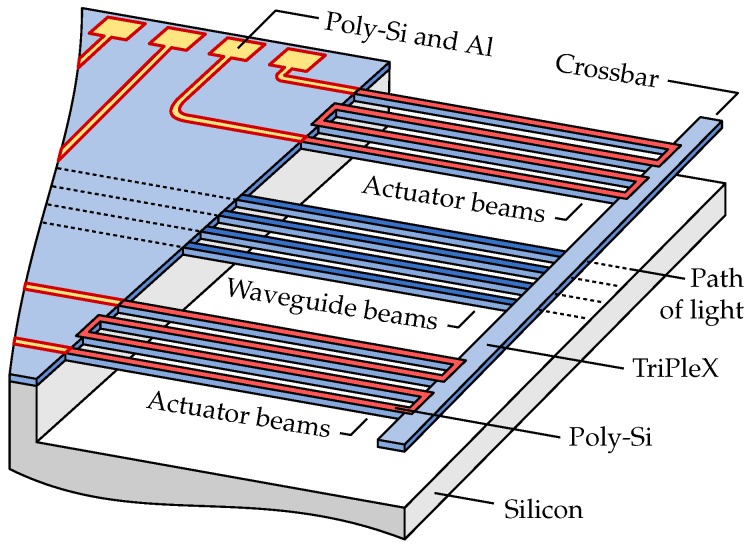
Schematic representation of a suspended positionable waveguide array with waveguide beams in the center and out-of-plane actuator beams on both sides.

For out-of-plane translation and rotation around the propagation direction of the light, a set of bimorph actuator beams is placed on both sides of the waveguide beams; see Figure 2. A crossbar is included in the design to connect the photonic waveguide beams and bimorph actuator beams at their free ends. In this way, all waveguide beams can be positioned by controlling only two actuators. One of the advantages of this approach is that the waveguide pitch, which is well-defined by lithography, is preserved. Moreover, a very small pitch can be realized, as no space is occupied by individual actuators between adjacent waveguide beams.

The waveguide beams are SiO${}_{2}$ beams that contain a waveguide core. The actuator beams are SiO${}_{2}$ beams with a patterned layer of poly-Si on top, as illustrated in Figure 2. Because the coefficient of thermal expansion (CTE) of poly-Si is larger than that of SiO${}_{2}$, the crossbar moves towards the silicon substrate when the actuator beams on both sides are operated simultaneously. In the presented designs, the poly-Si covers the full length of the actuator beams. In other work [11], we reported on bimorph beams that are partially covered by poly-Si, having the opportunity to, based on the design, control the post-release deformation, as is explained later.

The poly-Si material is both a structural part of the bimorph actuator and a heating element, by means of resistive heating. The doping level controls the resistivity of the poly-Si, and the resistivity together with the shape of the poly-Si heater (length and cross-section) determine its resistance. The actuators merge into electrical leads that terminate in bond pads. The electrical leads and bond pads consist of poly-Si with a layer of aluminum (Al) on top.

### 2.2. Dimensions

The configuration and dimensions of the positionable waveguide arrays follow from photonic, as well as mechanical requirements. An overview of the most important parameters, values and their rationale is given in Table 1. The number of waveguide beams is dependent on the number of channels of the photonic application. The cross-section of the waveguide beam is preferably as small as possible to ensure a low bending stiffness. The values for the waveguide thickness and width follow from the minimum required cladding thickness that ensures low propagation loss. The waveguide beam length is based on the required deflection and the force that thermal actuators can provide. The 127-μm pitch matches the pitch of commercially available fiber array units (FAUs). This pitch was chosen for practical convenience, and a smaller pitch (e.g., 50 μm) is feasible. A total of eight bimorph beams is included in the design (four on either side), placed at a pitch of 50 μm. The width of the crossbar is larger than the beam width to increase the crossbar stiffness. The crossbar is provided with etch holes to facilitate the release process. Two poly-Si thicknesses are selected, predominantly based on the resulting actuator stroke, as is discussed below. The waveguide core width together with the core thickness (which is not a parameter in the mask design, but defined by the Si${}_{3}$N${}_{4}$ thickness) determine the mode field diameter (MFD). At a wavelength of 1550 nm, 1 μm-wide and 200 nm-thin cores are single mode.

The waveguides in the bulk of the chip are designed to have an S-shape (visible on the surface of the interposer chip in Figure 1), to ensure that only the light that is coupled into the waveguide cores is guided to the positionable array.

**Figure 3:**
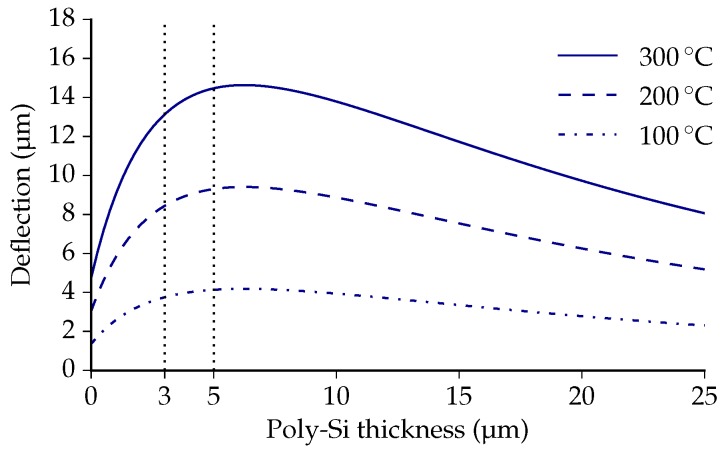
The deflection of the free end of an 800 μm-long, 18 μm-wide single bimorph beam as a function of the poly-Si thickness for a given SiO${}_{2}$ thickness of 16 μm. The three curves represent different actuator temperatures.

The actuator stroke of a single bimorph beam can be estimated using an analytical model, which is included in Appendix B. Figure 3 shows the free end deflection of an example bimorph beam as a function of the poly-Si thickness, for a given TriPleX thickness, at three different actuator temperatures. The free end moves towards the substrate upon actuation, and the maximum deflection is obtained with a poly-Si thickness that is slightly greater than 5 μm.

The initial, post-release deformation of the actuator beams is affected by the poly-Si thickness in a similar way. After the deposition of poly-Si at 1050 ${}^{\circ }C$, the temperature decreases to room temperature, introducing thermal stress. Inevitably, the bimorph effect causes a concave upward curvature of the actuator beams at room temperature. The maximum post-release curvature coincides with the maximum actuator stroke, at a poly-Si thickness just above 5 μm.

This paper aims to provide basic characteristics of SiO${}_{2}$/poly-Si bimorph actuators for universal use, rather than for a specific application only. To obtain a large deflection, two poly-Si thicknesses are selected, based on the curves in Figure 3: 3 and 5 μm. Furthermore, the bimorph actuators are designed to be fully covered by poly-Si, so that their performance is easily comparable with bimorph actuators based on different material configurations. These design choices are expected to result in a significant post-release deformation. Should the application require it, the post-release deformation can be reduced by means of alternative designs, e.g., partially covering bimorph beams with poly-Si [11].

## 3. Microfabrication

The suspended waveguide beams are released using a special reinforcement method, reducing the risk of beam fracturing. Without reinforcement, the compressive residual stress in the TriPleX layer causes the beams to buckle and fracture. Temporarily reinforcing the TriPleX beams with silicon (Si) reduces the expansion and buckling of suspended structures and prevents fracturing. More details on the reinforcement method and its working mechanism are available in previous work [9].

**Figure 4:**
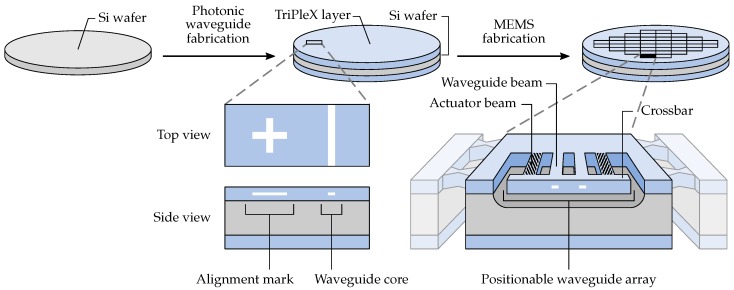
The two consecutive sequences for the fabrication of positionable waveguide arrays. First, photonic waveguide functionality is created. After that, MEMS functionality is realized.

The fabrication process of positionable waveguide arrays comprises two consecutive fabrication sequences, which are schematically illustrated in Figure 4. First, waveguiding functionality is added to a silicon wafer by depositing a TriPleX layer stack. This TriPleX stack predominantly consists of the following four different materials (from bottom to top): (1) ∼8 μm thick thermally-grown SiO${}_{2}$ bottom cladding layer; (2) 200 nm × ∼1 μm (thickness × width) silicon nitride (Si${}_{3}$N${}_{4}$) waveguide cores, grown by low pressure chemical vapor deposition (LPCVD); (3) ∼3 μm-thick LPCVD SiO${}_{2}$; and (4) ∼5 μm-thick SiO${}_{2}$ grown by plasma enhanced chemical vapor deposition (PECVD). Materials (3) and (4) together form the top cladding layer and are annealed at 1150 ${}^{\circ }C$ to bring their mechanical and optical properties close to those of thermally-grown SiO${}_{2}$. The Si${}_{3}$N${}_{4}$ layer includes alignment marks for the alignment of photolithography masks with the waveguide pattern.

**Figure 5:**
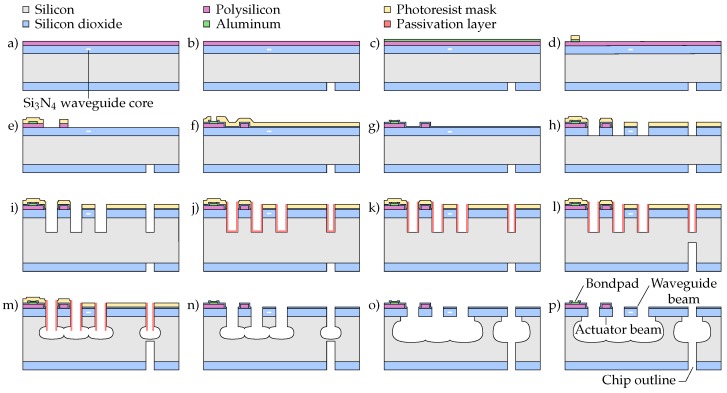
Fabrication steps for positionable suspended waveguide beam structures. (**a**) Deposition of poly-Si; (**b**) plasma etching of SiO${}_{2}$ on backside; (**c**) deposition of aluminum; (**d**) plasma etching of the aluminum; (**e**) plasma etching of the poly-Si; (**f**) deposition of PECVD SiO${}_{2}$ and local removal by plasma etching; (**g**) deposition of PECVD SiO${}_{2}$; (**h**) plasma etching of SiO${}_{2}$ on front-side; (**i**) anisotropic plasma etching of Si; (**j**) deposition of passivation layer; (**k**) local removal of passivation layer; (**l**) anisotropic plasma etching of Si on backside; (**m**) Isotropic plasma etching of Si; (**n**) removal of photoresist and passivation layer; (**o**) more isotropic etching of Si; (**p**) plasma etching of PECVD SiO${}_{2}$, to expose the bond pads.

After the waveguiding layer is formed, the SiO${}_{2}$ MEMS fabrication is performed, requiring a total of six lithography masks. The starting point of the MEMS fabrication is a 100 mm TriPleX wafer, resulting from the photonic waveguide fabrication, as is illustrated in Figure 4. The steps of the MEMS fabrication are as follows and are summarized in Figure 5.

On the front-side of the TriPleX wafer, poly-Si and aluminum are deposited (Figure 5a,c) and patterned (Figure 5d,e). On the backside, the SiO${}_{2}$ is locally removed by plasma etching (Figure 5b). Using two deposition steps and an etching step (Figure 5f,g), PECVD SiO${}_{2}$ is deposited with the objective to protect the poly-Si from being etched during the upcoming Si etch steps.

In order to define the beam structure, the SiO${}_{2}$ layer on the front-side is then patterned using plasma etching (Figure 5h). The silicon-reinforced release is performed by Steps i through m. First, using the same mask as was used for patterning the SiO${}_{2}$, the Si is anisotropically etched (Figure 5i). After depositing a passivation layer (Figure 5j), it is locally removed from the trench bottom. An anisotropic backside Si etch is then performed to define the chip outline (Figure 5l). The beam structure is then released by isotropically etching the Si on the front-side (Figure 5m). Once the complete structure is suspended, the photoresist and passivation layer are stripped (Figure 5n), followed by more isotropic etching of the Si (Figure 5o). Finally, electrical access to the bond pads is enabled by plasma etching the PECVD SiO${}_{2}$ (Figure 5p). More details of the microfabrication process are included in Appendix A.

**Figure 6:**
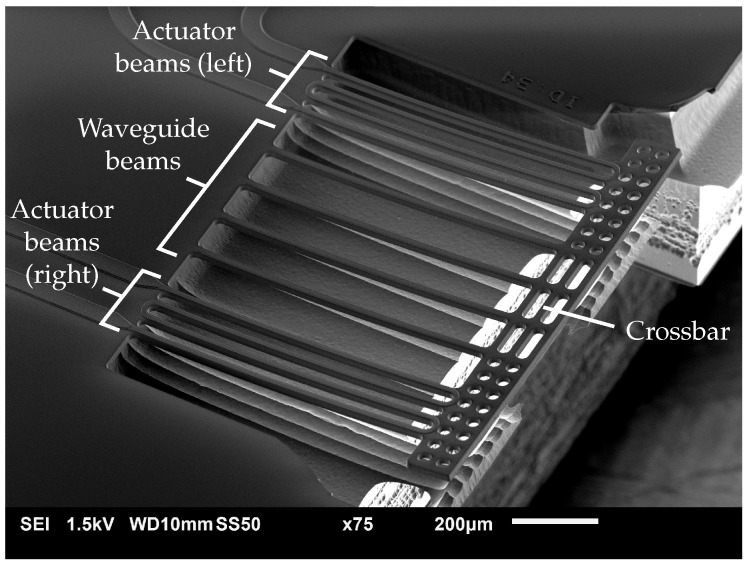
SEM image of a realized positionable waveguide array with a poly-Si thickness of 3 μm.

**Figure 7:**
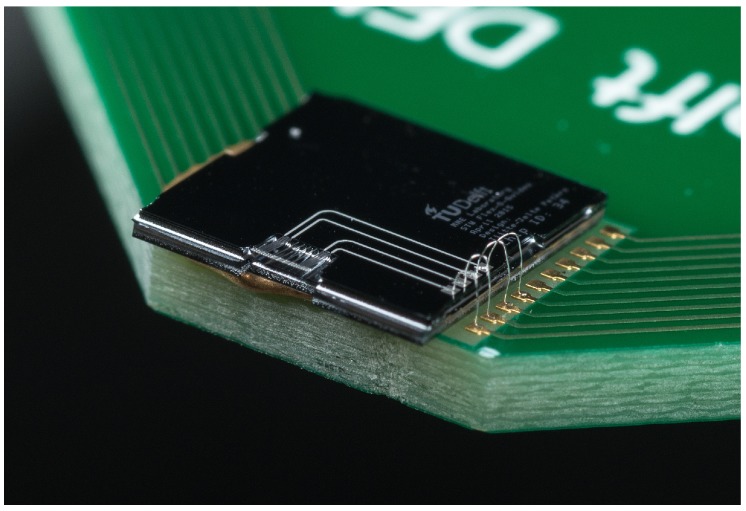
Photograph of a realized PIC with a positionable waveguide array, mounted on a PCB and electrically connected by wire bonding.

A realized positionable suspended waveguide array with 3 μm poly-Si thickness is presented in Figure 6. After singulating the PIC, it is mounted on a dedicated PCB and wire-bonded, as shown in Figure 7. Besides the 3 μm thickness variant, a positionable waveguide array with 5 μm-thick poly-Si is realized.

## 4. Experimental Results

This section describes the characterization of the realized suspended structures. The safe actuator voltage of 3 μm and 5 μm-thick poly-Si bimorph actuator beams is first determined. Furthermore, the post-release deformation, the deflection upon quasi-static actuation, the frequency response, the actuator force and the transient response of bimorph structures are measured. For all of these measurements, a comparison of the bimorph structures with 3 and 5 μm poly-Si thickness is presented.

### 4.1. Actuator Operating Range

A known issue with doped (poly-)Si actuators is the thermal runaway effect [12]. This effect can occur when a semiconductor heater is voltage-controlled. At a specific voltage regime, the resistive heating of the actuator causes the resistance of the poly-Si material to decrease. As a result, more current will flow through the actuator, heating it even more. If no limitation is set on the current, it will rapidly increase, resulting in the actuator to burn out. Lee et al. mention a typical thermal runaway temperature between 500 and 600 ${}^{\circ }C$ [13].

**Figure 8:**
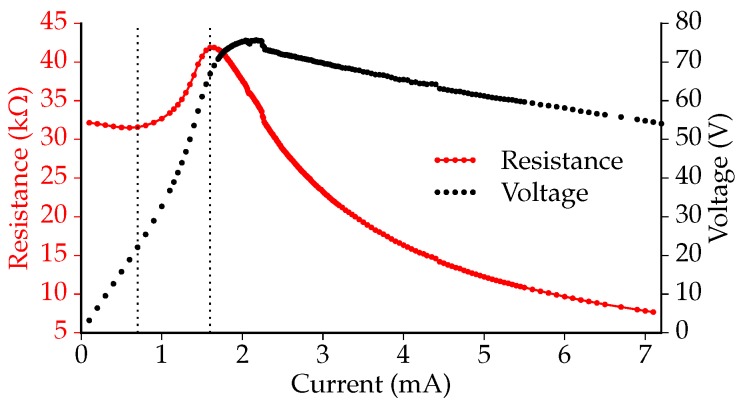
The measured resistance of and voltage over a sacrificial actuator (3 μm poly-Si thickness) as a function of the actuator current.

In order to identify the thermal runaway point and the breakdown point, the response of a sacrificial 3 μm poly-Si actuator to a current sweep was obtained using a 2611 system source meter (Keithley Instruments, Inc., Cleveland, OH, USA). Figure 8 shows the measured resistance, as well as the voltage of the sacrificial actuator as a function of the applied current. Three regimes can be distinguished in this graph, indicated with the vertical dotted lines. Between 0 and 0.7 mA, the resistance slightly decreases with increasing current. When the actuator current is between 0.7 and 1.6 mA, the resistance increases with the current. This phenomenon is known from other highly-doped poly-Si devices [14]. For 1.6 mA and higher, the resistance decreases as the actuator current increases. When voltage controlled, this is the regime where the thermal runaway would occur.

The measured voltage over the actuator increased from 0 to 75.5 V, after which, it decreased. Up to 7.2 mA could be applied to the actuator before it burnt out, corresponding to almost 400 mW of dissipated power. At an applied current of 3.7 mA (∼250 mW) and higher, the actuator emitted visible light.

**Figure 9:**
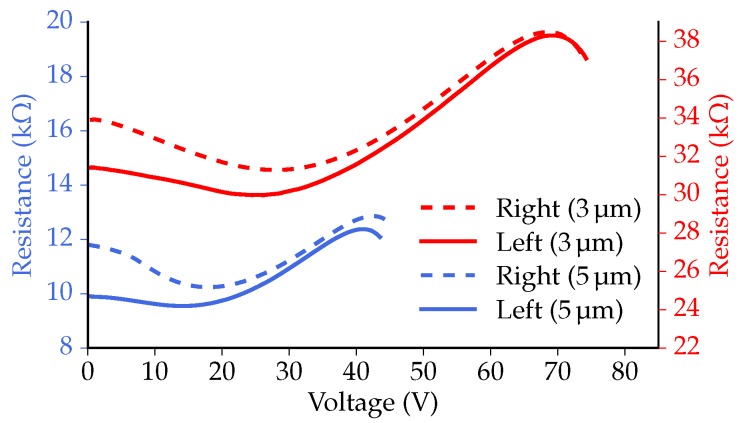
The results of current sweep measurements, showing the measured resistance of the actuators as a function of the measured voltage over the actuator.

To determine the safe operating range of the 3 and 5 μm-thick poly-Si actuators that will be analyzed below, comparable current sweep measurements are performed, with a maximum current just above the thermal runaway point. Figure 9 presents the measured change in resistance of the actuators on the left and right side of the two variants as a function of the measured voltage. The thermal runaway point is located around 70 V (∼130mW) for the 3 μm variant and around 40 V (∼125mW) for the 5 μm variant. Around the thermal runaway point, electrical resistances of approximately 38 kΩ and 13 kΩ were measured for the 3 μm and 5 μm variant, respectively. While there is a difference in voltage, caused by the difference in actuator resistance, the actuators show a comparable amount of power being dissipated around the thermal runaway points. The plot also shows that at low voltages, the resistance of the left actuators is slightly lower than that of the right actuators. We expect that this is due to the length difference of the poly-Si/Al electrical leads.

### 4.2. Post-Release Deformation

**Figure 10:**
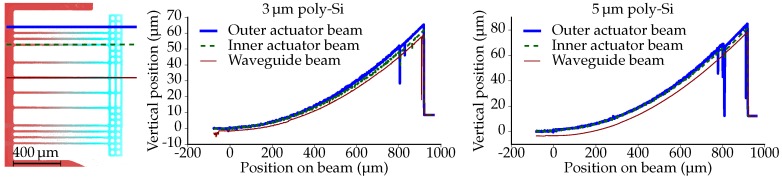
(**left**) Top view of the measured suspended structures, indicating the locations of the three profiles. (**middle**,**right**) The post-release deflection (without actuation) measured over the length of three beams.

The surface contours of suspended structures are obtained by means of white light interferometry (Contour GT-K 3D optical profilometer, Bruker Corporation, Billerica, MA, USA). The profiles measured over the length of one waveguide beam and two actuator beams are presented in Figure 10 for both variants (3 and 5 μm poly-Si thickness). For convenience, the vertical position of the base of the beams is aligned with the zero point of the vertical axis.

The beams have a post-release (i.e., without actuation) out-of-plane deflection. As a result, the free ends of the beams are located approximately 60 μm and 80 μm above the surface of the PIC, for the 3 μm and 5 μm poly-Si thickness, respectively. The curvatures of the three measured beams range from 126142/m for the 3 μm poly-Si structure and from 180204/m for the 5 μm poly-Si structure.

**Figure 11:**
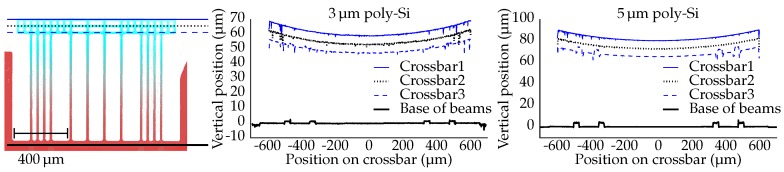
(**left**) Top view of the measured suspended structures, indicating the locations of the four profiles. (**middle**,**right**) The post-release deflection (without actuation) measured over the length of the crossbar and at the base of the beams.

Figure 11 presents the profiles as measured on three locations on the crossbars and at the base of the beams. The profiles at the base of the beams reveal elevated regions, corresponding to the poly-Si/Al pattern. Furthermore, these profiles indicate that the PIC surfaces are flat. For convenience, the vertical position of the surface of the PICs is aligned with the zero point of the vertical axis. The profiles measured over the length of the crossbars reveal that the crossbars have a concave upward curvature, corresponding to approximately 20/m. There are two effects that influence the curvature of the crossbar. Firstly, the curvature of the bimorph beams is larger than that of the waveguide beams; secondly, the crossbar itself has a post-release curvature due to residual stress in the various oxide layers. In previously-fabricated suspended SiO${}_{2}$ structures without actuators, this curvature was measured to be ∼87/m [15].

### 4.3. Actuation: Bimorph Actuators

The actuators are characterized by operating them with the Keithley 2611 system source meter, while measuring their deflection with the white light interferometric profilometer. The bimorph actuators on both sides of the waveguide beams are electrically connected in parallel, and the out-of-plane deflection of the crossbar is measured at different voltage levels. To prevent the thermal runaway effect, a very safe power limit of 60 mW per actuator was imposed.

In order to obtain high quality deflection data of the narrow beams, the profilometer was operated using a 20× magnification. As a consequence, covering the full crossbar surface required multiple images to be recorded and stitched. A full crossbar measurement takes roughly 90 s, during which the voltage over the actuators is kept constant. Over this time span, the deflection can be assumed to be constant. This is confirmed by a stability experiment of a positionable waveguide array with a smaller pitch (50 μm), revealing a 0.16 μm drift in 5 min with approximately 50 mW actuation power [16].

**Figure 12:**
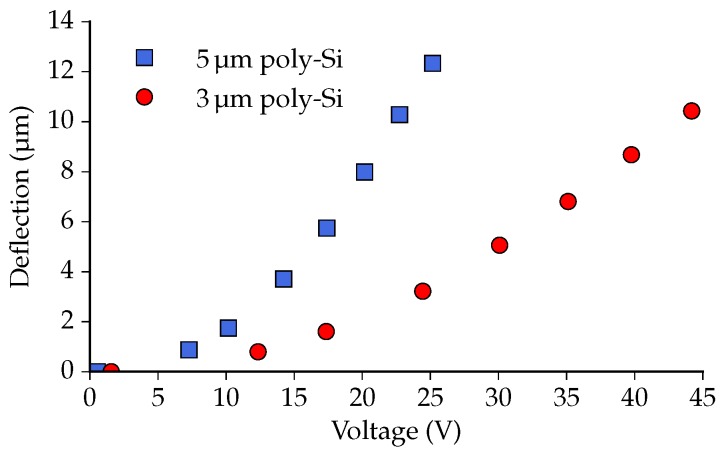
The vertical deflection of the center of the crossbar as a function of the actuator voltage.

Figure 12 presents the out-of-plane deflection of the center of the crossbars as a function of actuator voltage. The 3 μm actuator achieves a 10.4 μm vertical deflection (towards the chip substrate) when 44.2 V is applied. The 5 μm variant delivers 12.3 μm out-of-plane motion with an actuator voltage of 25.2 V. This difference in voltage is caused by the difference in actuator resistance. When the left side and right side are connected in parallel, the actuators have a mean resistance of ∼15.4 kΩ and ∼5.2 kΩ, for the 3 μm and 5 μm actuators, respectively.

**Figure 13:**
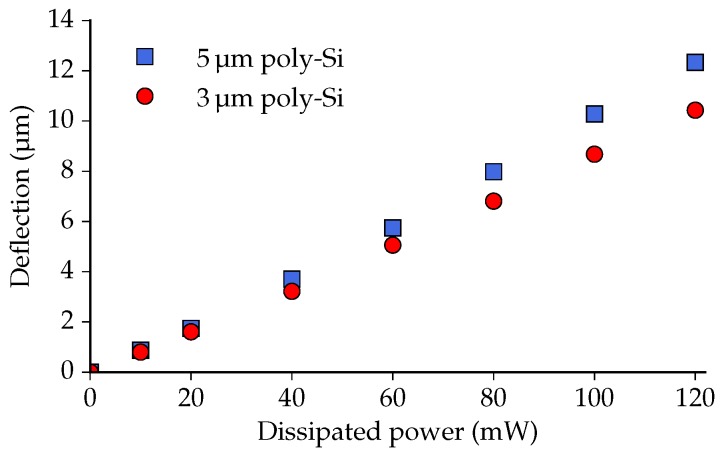
The vertical deflection of the center of the crossbar as a function of the power dissipated in the two actuators.

Figure 13 shows the out-of-plane deflection of the crossbars as a function of the total power applied to the two actuators. It can be seen that the deflection of the 5 μm version is larger than that of the 3 μm version for a given amount of dissipated power. With 120 mW power applied, for example, the 5 μm-thick poly-Si achieves an ∼1.9 μm (or ∼18 ) larger deflection than the 3 μm variant.

**Figure 14:**
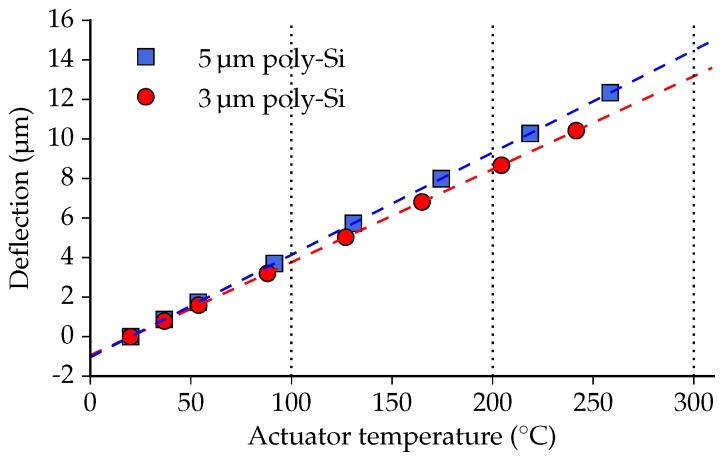
The vertical deflection as a function of the estimated actuator temperature. The dashed lines represent linear functions fitted to the data points. As a visual guide, 100 °C, 200 °C and 300 ${}^{\circ }C$ are indicated by dotted lines.

For an estimation of the mean actuator temperature, the change in deflection of single actuator beams due to a temperature difference is determined using the analytical model in Appendix B. The analytical model shows that the deflection of the free end of a trilayer beam changes linearly with the temperature: 47 $nm{/}^{\circ }C$ and 52 $nm{/}^{\circ }C$ for the 3 μm and 5 μm poly-Si thickness, respectively. Using this linear relationship, the deflection can be plotted as a function of the estimated actuator temperature, as is presented in Figure 14.

The data points in Figure 14 correspond well with their linear fits, which is in agreement with the linear temperature dependence of the deflection following from the analytical model. Comparing the deflection values at 100 °C, 200 °C and 300 ${}^{\circ }C$ with the analytical results presented in Figure 3, they match quite well. The temperature of the integrated actuators will be slightly higher than the estimated temperature, because deflecting a positionable array (consisting of both actuator beams and waveguide beams) requires more force than deflecting a single actuator beam. Nevertheless, the analytical single beam model is a good estimator for the vertical deflection of this positionable waveguide array. Additionally, based on this temperature estimate, it is safe to assume that the actuator temperature stays well below the recrystallization temperature of poly-Si, which is around 600 ${}^{\circ }C$ [17].

### 4.4. Frequency Response

**Figure 15:**
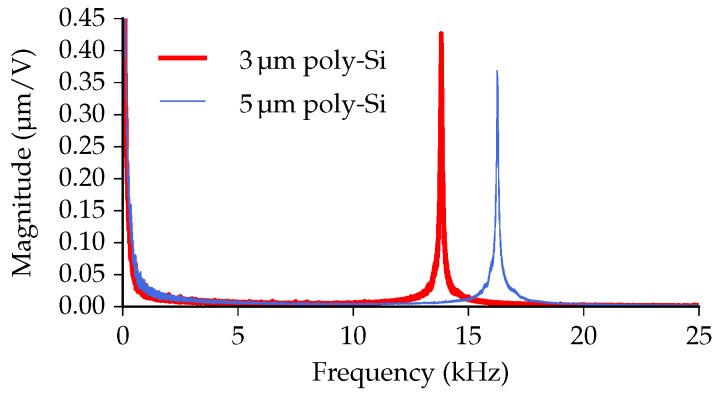
The frequency response of the positionable waveguide arrays from 025 kHz. The measured resonance peaks are at 13.8 kHz and 16.2 kHz.

The frequency response of the positionable waveguide arrays is measured using an MSA-400 scanning vibrometer (Polytec Inc., Waldbronn, Germany) and presented in Figure 15. From 0 kHz to 25 kHz, only one resonance peak was observed for both systems. A resonance peak at 13.8 kHz was measured for the 3 μm poly-Si variant, while the 5 μm poly-Si system has a peak at 16.2 kHz, both corresponding with the first vertical resonance mode. The 5 μm poly-Si array, having a larger thickness than the 3 μm variant, has a higher out-of-plane stiffness. As a result, although the length and width are the same, the resonance frequency of the 5 μm system is higher than that of the 3 μm system.

### 4.5. Bending Stiffness and Actuator Force

The out-of-plane bending stiffness of the array can be approximated using the measured resonance frequency. The first mode frequency $f_{0}$ and the out-of-plane stiffness *k* are linked by the relation:

(1) $$f_{0}=\frac{1}{2\pi }\sqrt{\frac{k}{m_{\mathrm{crossbar}}+\frac{33}{140}m_{\mathrm{beams}}}},$$

where $m_{\mathrm{crossbar}}$ is the mass of the crossbar and $m_{\mathrm{beams}}$ is the mass of the waveguide and actuator beams [18]. The measured waveguide arrays consist of four waveguide beams (SiO${}_{2}$ only) and eight actuator beams (SiO${}_{2}$ with poly-Si). Appendix C provides the calculations of the mass of the crossbar and the two types of beams. The out-of-plane stiffness is calculated to be $k_{3\mu m}=44N/m$ and $k_{5\mu m}=62N/m$.

The produced actuator force is determined by the stiffness and the deflection of the arrays. The 3 μm system produces a force of about $F_{3\mu m}=k_{3\mu m}\;\delta y=458\mu N$, based on a measured vertical crossbar deflection δy=10.4μm. The 5 μm system produces a force of about $F_{5\mu m}=k_{5\mu m}\;\delta y=757\mu N$, considering a crossbar deflection of δy=12.3μm.

### 4.6. Transient Response

**Figure 16:**
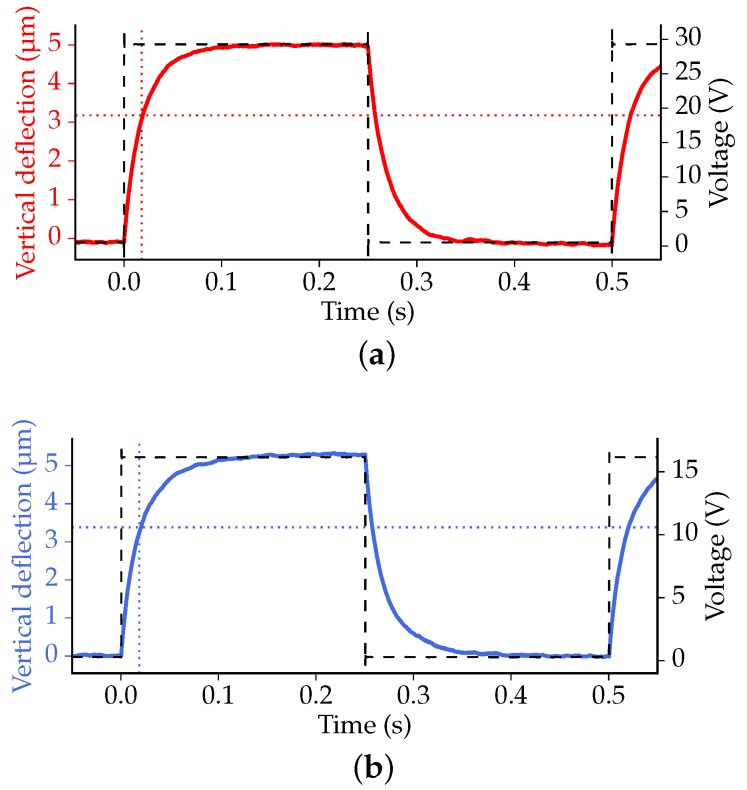
The vertical deflection (solid line) of a point on the crossbar resulting from an applied square wave voltage (dashed line). The plots indicate the 63.2% level (horizontal dotted line) and the corresponding time value (vertical dotted line). (**a**) Actuators with 3 μm poly-Si thickness; (**b**) actuators with 5 μm poly-Si thickness.

The transient thermal response of the actuators is explored, again using the Polytec MSA-400. By measuring the position of the free end of the actuator beams, the response to switching the state of the actuator (on/off) can be obtained. The out-of-plane displacements induced by an applied low frequency (2 Hz) square wave are presented in Figure 16a (3 μm actuators) and Figure 16b (5 μm actuators). An amplitude of 29V was used for the 3 μm actuators and 16V for the 5 μm actuators in order to obtain a deflection close to 5 μm.

According to the measurements, the vertical deflection changes exponentially with time. The thermal response of the bimorph actuators is governed by heat transfer taking place predominantly through convection and conduction, since the effect of radiation is negligible at the scale of the actuators. A steady temperature distribution is obtained after roughly 0.1 s, for the two types of tested actuators. The 63.2% time constant of the 3 μm variant was found to be 18 ms. For the 5 μm system, the 63.2% time constant was measured to be 19 ms. No significant difference between rise and fall times was observed, indicating that the time required for heating and cooling is about the same.

## 5. Discussion and Conclusions

The fabricated positionable waveguide arrays have a curved crossbar, which introduces a vertical misalignment in case more than two waveguides need to be aligned with two other waveguides. This crossbar curvature can be prevented by adjustments in the design/fabrication. By covering the waveguide beams with a layer of poly-Si similar to the layer on the actuator beams, all beams will have the same curvature. The consequence of this will be that the total structure becomes stiffer, and a smaller deflection will be obtained with the same actuators. Another cause for the crossbar curvature is residual stress, resulting from the PECVD SiO${}_{2}$ layer that is deposited on top of the TriPleX crossbar. In the presented designs, the PECVD SiO${}_{2}$, which aims to protect the poly-Si from being etched, is exclusively removed at the bond pad locations. By also removing the PECVD SiO${}_{2}$ layer from the crossbar (except where it is needed to protect poly-Si), the crossbar will not be curved by residual stress in the PECVD SiO${}_{2}$/TriPleX combination.

The bimorph actuators are measured to provide a downward deflection of approximately 11 μm when operated simultaneously at 60 mW per actuator side. The expected maximum deflection that can be obtained, based on extrapolation of the measured data, is about two times as large, as the actuators can be driven to approximately 125 mW before reaching the thermal runaway point.

The analytical model and the experimental results reveal that the design of the bimorph beams affects the actuator stroke and the post-release deflection in a similar way. For example, it was shown that a poly-Si thickness that optimizes the stroke also maximizes the post-release deformation. The coupling between the post-release deformation and the actuator stroke can be conflicting for some applications.

For the alignment concept, a post-release deformation in the order of 10 μm and an actuator stroke of about 4 μm would be ideal. The 10 μm post-release deformation ensures that, when the two PICs are flip-chip bonded, the opposing waveguide cores have an appropriate initial vertical offset, such that driving the actuators reduces this offset. The 4 μm actuator stroke is sufficient to compensate any vertical misalignment introduced in the pre-alignment. The positionable waveguide array design as presented in this paper results in an actuator stroke that is more than sufficient to perform the fine-alignment, whereas the post-release deformation is about seven-times larger than required. As a result, the post-release deformation causes the crossbar to have a vertical offset of approximately 70 μm with respect to the waveguides of the PIC, preventing a decent photonic alignment.

Actuator design improvements can be implemented to make the bimorph actuators better suited for specific applications. For example, covering only a particular region of the actuator beams with poly-Si significantly reduces the post-release deformation, while the actuator stroke only decreases slightly [11]. Another approach to adjusting the post-release deformation is by controlling the stress and the stress gradient within the poly-Si layer, which is demonstrated in [19]. For certain applications, the associated increase in complexity of the fabrication process might well be a justifiable investment.

This paper presents a thorough characterization of the vertical (out-of-plane) translation of the positionable waveguide arrays, based on driving both actuator sides simultaneously. By actuating one actuator side more than the other, the crossbar can be moved downward while it simultaneously is slightly rotated around the propagation direction of the light. This kind of actuation is beneficial for the photonic alignment concept, in the case of rotational misalignment resulting from the pre-alignment stage. Moreover, the photonic alignment concept will require horizontal (in-plane) translation of the positionable waveguide array, as acceptable coupling loss between the photonic waveguides can only be attained with submicrometer in-plane alignment precision. Rotational and in-plane actuation functions are currently under investigation, and results will be published once they are available.

The experiments revealed a difference in the resistance of the actuators on the left and the right side, probably due to the length difference of the electrical leads. Driving both actuators with an equal voltage might result in an imbalance in the deflection of both sides. Regarding the photonic alignment, this resistance dissimilarity is not expected to be problematic, because the actuators will be driven independently and active alignment will be applied to obtain the optimal alignment. For applications that do not provide any feedback on the position, the electrical leads are best designed to have equivalent resistance values.

No strict requirements are imposed on the time constant, in the case that the bimorph actuators are utilized for fine-aligning purposes, as in the alignment concept. For other SiO${}_{2}$ MEMS applications, however, it can be beneficial to have a small time constant. With 63.2% of time constants smaller than 20 ms, both the 3 μm and 5 μm system are able to switch between 0 μm and 5 μm vertical deflection roughly five times per second. When operated at the resonance frequency (13.8 kHz and 16.2 kHz), even higher frequencies can be achieved.

In conclusion, this paper presented the integration of electrothermal actuators with a photonic material platform that predominantly consists of thick SiO${}_{2}$. Using a special silicon reinforcement method, fracturing was prevented, and suspended SiO${}_{2}$ beam structures were fabricated. Positionable arrays of photonic waveguides were realized by integrating thermal out-of-plane bimorph actuators. The actuators were created using two different poly-Si thicknesses on top of SiO${}_{2}$ beams, enabling the comparison of the different poly-Si thickness variants.

The positionable waveguide arrays have a significant post-release out-of-plane deflection. The crossbars of the arrays with a beam length of 800 μm are located 60 μm and 80μm above the chip surface of the 3 μm and 5 μm variant, respectively.

The bimorph actuators, when operated simultaneously, provide an out-of-plane deflection of the crossbar of approximately 11 μm at 60 mW dissipated power per actuator side, corresponding to a temperature in the order of 240 ${}^{\circ }C$. Analytical model results of single bimorph beams give a reasonable estimate of positionable waveguide array deflections. The actuators deliver an estimated force of 450 μN and 750μN for the 3 μm and 5 μm poly-Si thickness, respectively. The time constants were found to be 18 ms for the 3 μm and 19 ms for the 5 μm variant. By adjusting the design of the actuators, their performance can be tuned to meet application-specific requirements.

multiple

## Figures and Tables

**Table 1:** Overview of the most important parameters and values used in the suspended beam arrays. The rationale column indicates whether the value is defined by photonic (p) or mechanical (m) requirements.

Parameter	Value	Rationale
Number of waveguide (WG) beams	4	p
WG beam thickness (μm)	16	p + m
WG beam width (μm)	18	p + m
WG beam length (μm)	800	m
WG pitch (μm)	127	p
Number of bimorph beams	8	m
Bimorph beam pitch (μm)	50	m
Crossbar width (μm)	118	m
Poly-Si thickness (μm)	3 or 5	m
WG core width (μm)	1	p
WG core thickness (nm)	200	p

**Figure B1:**
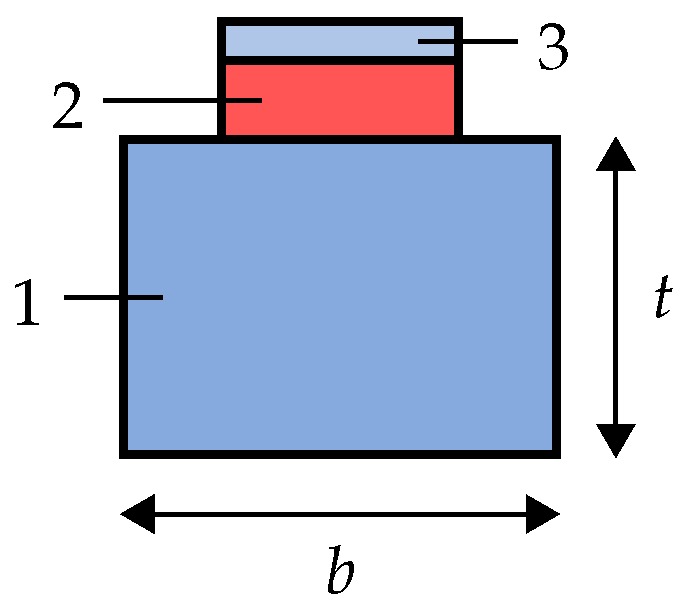
The cross-sectional dimensions of the three layers representing a bimorph beam.

**Figure C1:**
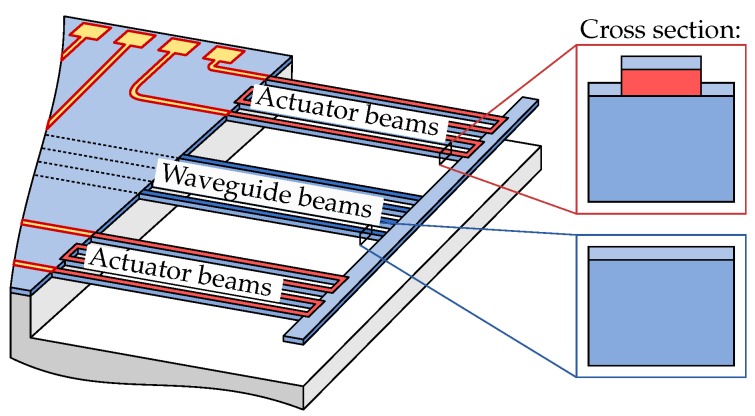
The layout of the measured arrays, indicating the cross-sections of the waveguide beams and the actuator beams.

## Appendix A. Microfabrication Process

This Appendix provides the details of the microfabrication process. Reference is made to the process steps as depicted in Figure 5 in Section 3.

A 130 nm thin seed layer of LPCVD poly-Si is deposited on the TriPleX wafer. To prevent poly-Si accumulation on the backside in the consecutive epitaxial growth step, the poly-Si is removed from the backside of the wafer. This etching step is completed without a mask in a Trikon Omega 201 plasma system. The etch recipe consists of a SiO${}_{2}$ breakthrough followed by a Si etch, both performed using an inductively coupled plasma (ICP) power of 500 W at a temperature of 20 ${}^{\circ }C$. The SiO${}_{2}$ breakthrough uses 20 sccm of O${}_{2}$ and 40 sccm of CF${}_{4}$, a pressure of 5 mTorr and a bias RF power of 60 W. The Si etch uses 80 sccm of Cl${}_{2}$ and 40 sccm of HBr, a pressure of 60 mTorr and a bias RF power of 20 W.

Immediately before the epitaxial growth, native oxide is removed by wet etching in a solution with 0.55% hydrogen fluoride (HF) for 4 min. During the epitaxial growth, the thickness of the poly-Si seed layer is increased. The final thickness is controlled by the duration of the epitaxial growth process, taking into account the growth rate (i.e., approximately 1 μm/min). The epitaxial growth process is performed at a temperature of 1050 ${}^{\circ }C$, a pressure of 60 and with a flow of diborane (B${}_{2}$H${}_{6}$) diluted with hydrogen gas (H${}_{2}$) to control the p-type doping level of the poly-Si layer. A source/inject/diluent ratio of 110 sccm/65 sccm/20 SLM was applied, aiming for a doping concentration equal to 2 × 10^16^ $B/{\mathrm{cm}}^{3}$ in crystalline Si. The resulting cross-section is depicted in Figure 5a.

Locally, rectangular openings are plasma etched in the poly-Si layer to regain visual access to the alignment marks. SPR3012 positive resist (MicroChem Corp., Westborough, MA, USA) with a thickness of 1.4 μm is used as the etch mask. A Bosch process is employed by means of an Omega i2L Rapier deep silicon etcher (SPTS Technologies Ltd., Newport, Wales). This process continually cycles through the deposition of a passivation layer and two etch steps. The deposition step has a duration of 1.2 s and uses C${}_{4}$F${}_{8}$ gas without platen HF power at a chamber pressure of 40 m. The first etch step lasts 1.2 s, using O${}_{2}$ gas and 75 W platen HF power. The second etch step is 2.5 s long and uses SF${}_{6}$ gas with 24 W platen HF power. Both etch steps use 30 m chamber pressure. The deposition, as well as the etch steps are performed with 2500 W primary source power and 500 W secondary source power.

Next, the desired singulation pattern is etched into the SiO${}_{2}$ layer on the backside of the wafer; see Figure 5b. This backside processing step is performed at this stage of the fabrication process in order to prevent possible damage to the future structures at the front-side. The etching is performed with a 6 μm-thick etching mask (positive AZ9260 resist, MicroChemicals GmbH, Ulm, Germany) and a Drytek Triode T384 system using C${}_{2}$F${}_{6}$ and CHF${}_{3}$ gasses. The gas flows are 36 sccm and 144 sccm, respectively, and an RF power of 300 W is used at 180 m chamber pressure. After removing the photoresist using an oxygen plasma, the processing continues on the front-side.

On top of the poly-Si layer, a 675 nm thin layer of Al with 1 of Si (Al–1%Si) is deposited by sputter deposition (Figure 5c). Al–1%Si is used instead of pure Al to prevent Al spikes in the Si due to Si–Al interaction at elevated temperatures. The deposition is performed with a Trikon Sigma 204 sputter coater, at 350 ${}^{\circ }C$, using a base pressure of 1 × 10^−7^ Torr and 10 kW target power. Locally, rectangular openings are plasma etched in the Al–1%Si layer to regain visual access to the alignment marks. The Trikon Omega 201 plasma system is used for this etching step, applying 30 sccm of chlorine gas (Cl${}_{2}$), 40 sccm of hydrogen bromide gas (HBr), 5 m process pressure, 40 W platen RF power and 500 W ICP RF power.

With the alignment marks visible, a 1.4 μm-thick SPR3012 positive resist layer is spin coated, and its pattern is used as the mask during the etching of the Al–1%Si, forming the electrical leads and the bond pads, which is illustrated in Figure 5d. The etching of Al–1%Si is done in the Trikon Omega 201 plasma system, employing a plasma etch process with HBr (40 sccm) and Cl${}_{2}$ (30 sccm) at a process pressure of 5 mTorr. The etching is performed in two steps, first applying 50 W platen RF power and 500 W ICP RF power for 25 s and, subsequently, 40 W platen RF power and 500 W ICP RF power for 40 s.

A 4 μm-thick layer of SPR3017M photoresist (MicroChem Corp.) is then spin coated, covering the >0.675 μm topography of the Al–1%Si and poly-Si. The pattern in this mask includes the electrical leads and bond pads, but also defines the poly-Si heating structures as follows. The combination of poly-Si and Al–1%Si is used for electrical conductance, whereas poly-Si without Al–1%Si serves as a heater. For the etching of the poly-Si, the same recipe was used on the SPTS Omega i2L Rapier deep silicon etcher as in the step in which the openings were etched in the poly-Si. The resulting cross-section is presented in Figure 5e.

A 2 μm-thick layer of SiO${}_{2}$ is deposited by plasma-enhanced chemical vapor deposition (PECVD). This layer is applied to prevent the poly-Si from being etched during the upcoming Si etch steps. The PECVD SiO${}_{2}$ is deposited with a Novellus Concept One system, using a temperature of 400 ${}^{\circ }C$, a pressure of 2.2 Torr and an HF power of 1000 W. Gas flows of 205 sccm for SiH${}_{4}$, 6000 sccm for N${}_{2}$O and 3150 sccm for N${}_{2}$ are used. After spin coating and patterning a 4 μm layer of Shipley SPR3027 photoresist (MicroChem Corp.), the PECVD SiO${}_{2}$ is locally etched where bond pads will be realized (shown in Figure 5f). For the SiO${}_{2}$ etching, the Drytek Triode T384 system is used again, with similar settings.

Although finally, the Al bond pads are required to be accessible for making electrical connections, they must be protected from being etched during the intermediate process steps. For that reason, another layer of PECVD SiO${}_{2}$, this time 0.3 μm in thickness, is deposited, as depicted in Figure 5g. The Novellus Concept One system is used with similar temperature, pressure, gas flows and power, but shorter deposition time.

Photoresist (AZ9260) is spin coated with a thickness of 12 μm and patterned into a photoresist mask. A thickness of 12 μm is used for two reasons. Firstly, the topography that has to be covered is significant, with height variations up to 7 μm; secondly, this mask is used during multiple etching steps, one of which is a relatively long etch through ∼18 μm of SiO${}_{2}$. The front-side SiO${}_{2}$ is etched using the Drytek Triode T384 system with the same parameters as used for the etching of the singulation pattern on the backside. Figure 5h shows the cross-section resulting from this etch. This etching step not only defines the beam structures that will be suspended, but also the chip outline on the front-side, including supports on the corners of every chip (as indicated in Figure 4).

After that, the SPTS Omega i2L Rapier deep silicon etcher is used to perform consecutive steps, applying SF${}_{6}$ for etching and C${}_{4}$F${}_{8}$ for passivation layer deposition. (1) Without removing the photoresist mask, the same pattern is anisotropically plasma etched in the Si (Figure 5i); (2) a CF${}_{X}$ passivation layer with approximately 1 μm thickness is deposited (Figure 5j) and, then; (3) locally removed from the trench bottom (Figure 5k); (4) from the backside, Si is etched using the SiO${}_{2}$ as a mask (Figure 5l); (5) the unprotected Si is now isotropically plasma etched, until the complete structure of Si-reinforced SiO${}_{2}$ is suspended (Figure 5m); after (6) complete removal of the photoresist and passivation layer (Figure 5n) in an O${}_{2}$ plasma (Tepla 300 plasma system); (7) more isotropic etching of Si leads to suspended SiO${}_{2}$ structures without Si reinforcement (Figure 5o).

In the final step, ∼0.3 μm of PECVD SiO${}_{2}$ is etched with an Alcatel GIR300 Fluorine etcher (Alcatel, Annecy, France), enabling electrical access to the Al bond pads; see Figure 5p. Gas flows of 50 sccm for CF${}_{4}$, 25 sccm for CHF${}_{3}$ and 40 sccm for He are used at a pressure of 0.05 mbar and with 60 W power.

## Appendix B. Analytical Multilayer Beam Model

Based on the analytical multilayer beam model as described by Scott et al. [20], the curvature of a trilayer cantilever is:

$$\kappa =\frac{\left(2RA^{-1}S\right)}{2+RA^{-1}B},$$

with:

$$R=\left[\begin{array}{c} \frac{t_{1}}{2}\\ t_{1}+\frac{t_{2}}{2}\\ t_{1}+t_{2}+\frac{t_{3}}{2} \end{array}\right]\frac{-1}{E_{1}I_{1}+E_{2}I_{2}+E_{3}I_{3}},$$

$$A=\left[\begin{array}{ccc} {\left(E_{1}A_{1}\right)}^{-1} & -{\left(E_{2}A_{2}\right)}^{-1} & 0\\ 0 & {\left(E_{2}A_{2}\right)}^{-1} & -(E_{3}A_{3}^{-1})\\ 1 & 1 & 1 \end{array}\right],$$

$$S=\left[\begin{array}{c} \epsilon_{2}-\epsilon_{1}\\ \epsilon_{3}-\epsilon_{2}\\ 0 \end{array}\right],$$

$$B=\left[\begin{array}{c} t_{1}+t_{2}\\ t_{2}+t_{3}\\ 0 \end{array}\right],$$

$$I_{i}=\frac{b_{i}t_{i}^{3}}{12},$$

$$A_{i}=b_{i}t_{i},$$

$$\epsilon_{i}=\alpha_{i}\Delta T,$$

and where $b_{i}$, $t_{i}$, $E_{i}$, $\alpha_{i}$ are the width, thickness, modulus of elasticity and the CTE of layer *i*, respectively. ΔT is the applied temperature difference. This model considers thermal stress only.

The free end deflection *δ* of a beam with curvature *κ* can be approximated by:

$$\delta =\frac{\left(1-\cos\kappa L\right)}{\kappa },$$

where *L* is the length of the beam (800 μm in our case). For a given length and thickness of the three layers, the deflection of the free end is found to be a linear function of the actuator temperature. The deflection results depicted in Figure 3 are based on the layer stack as presented in Figure B1 and the width (*b*), thickness (*t*), Young’s Moulus (*E*), and CTE (*α*) of every layer as summarized in Table B1. For the TriPleX layer, which is a combination of SiO${}_{2}$ and Si${}_{3}$N${}_{4}$, the values for *E* and *α* are assumed to be close to those of SiO${}_{2}$. On a total thickness of ∼16 μm, the SiO${}_{2}$ thickness is dominant, whereas the Si${}_{3}$N${}_{4}$ thickness is in the order of 200 nm. Moreover, the width of the Si${}_{3}$N${}_{4}$ waveguide core is approximately 17 μm smaller than the total beam width.

**Table B1:** The values of the trilayer materials.

Layer	1	2	3
Material	TriPleX	Poly-Si	SiO${}_{2}$
*b* (μm)	18	12	12
*t* (μm)	16	3 or 5	2
*E* (GPa)	70	160	75
*α* ($10^{-6}{/}^{\circ }C$)	0.5	3.44	2.5

## Appendix C. Determining the Mass

The out-of-plane bending stiffness of the complete system is calculated using 1, which requires the mass of the beams and the crossbar. Figure C1 presents the layout of the positionable waveguide array. The total system comprises eight actuator beams, four waveguide beams and a crossbar. The mass of the beams is approximated as follows, using the values from Table B1.

### Appendix C.1. Waveguide Beams

A single waveguide beam consists of two layers. The bottom layer has a width of 18 μm, a thickness of 16 μm and a density of 2200 $kg/m^{3}$. The top layer has a width of 18 μm, a thickness of 2 μm and a density of 2300 $kg/m^{3}$. Furthermore, the beam length is 800 μm. The mass of a single waveguide beam $m_{\mathrm{wg}-\mathrm{beam}}$ is:

$$\begin{array}{cc} m_{\mathrm{wg}-\mathrm{beam}} & =\rho_{1}V_{1}+\rho_{3}V_{3}\\ & =\rho_{1}Lb_{1}t_{1}+\rho_{3}Lb_{3}t_{3}\\ & =2200kg/m^{3}\times 800\;\mu m\times 18\;\mu m\times 16\;\mu m+\\ & \;2300kg/m^{3}\times 800\;\mu m\times 18\;\mu m\times 2\;\mu m\\ & \approx 5.73\times 10^{-7}kg \end{array}$$

### Appendix C.2. Actuator Beams

The mass of the actuator beams depends on the poly-Si thickness. A single actuator beam consists of the same two layers comprising a waveguide beam, with an extra layer of poly-Si. The poly-Si layer has a width of 12 μm, a thickness of 3 μm and a density of 2320 $kg/m^{3}$. Again, a beam length of 800 μm is used. The mass of a single 3 μm actuator beam $m_{\mathrm{act}-\mathrm{beam},3}$ is:

$$\begin{array}{cc} m_{\mathrm{act}-\mathrm{beam},3} & =m_{\mathrm{wg}-\mathrm{beam}}+\rho_{2}V_{2}\\ & =m_{\mathrm{wg}-\mathrm{beam}}+\rho_{2}Lb_{2}t_{2}\\ & =m_{\mathrm{wg}-\mathrm{beam}}+2320kg/m^{3}\times 800\;\mu m\times 12\;\mu m\times 3\;\mu m\\ & =5.73\times 10^{-7}kg+6.68\times 10^{-8}kg\\ & \approx 6.40\times 10^{-7}kg \end{array}$$

The mass of a single 5 μm actuator beam $m_{\mathrm{act}-\mathrm{beam},5}$ is:

$$\begin{array}{cc} m_{\mathrm{act}-\mathrm{beam},5} & =m_{\mathrm{wg}-\mathrm{beam}}+\rho_{2}V_{2}\\ & =m_{\mathrm{wg}-\mathrm{beam}}+\rho_{2}Lb_{2}t_{2}\\ & =m_{\mathrm{wg}-\mathrm{beam}}+2320kg/m^{3}\times 800\;\mu m\times 12\;\mu m\times 5\;\mu m\\ & =5.73\times 10^{-7}kg+1.11\times 10^{-7}kg\\ & \approx 6.84\times 10^{-7}kg \end{array}$$

### Appendix C.3. Crossbar

The crossbar consists of the same two layers comprising the waveguide beams. The crossbar has a width of 118 μm and a length of 1200 μm. By reducing the width of the crossbar with 32 μm, the loss of mass due to the etch holes is compensated, resulting in an effective crossbar width of 86 μm. The mass of the crossbar $m_{\mathrm{cb}}$ is:

$$\begin{array}{cc} m_{\mathrm{cb}} & =\rho_{1}V_{1}+\rho_{3}V_{3}\\ & =\rho_{1}Lb_{1}t_{1}+\rho_{3}Lb_{3}t_{3}\\ & =2200kg/m^{3}\times 1200\;\mu m\times 86\;\mu m\times 16\;\mu m+\\ & \;2300kg/m^{3}\times 1200\;\mu m\times 86\;\mu m\times 2\;\mu m\\ & \approx 4.11\times 10^{-6}kg \end{array}$$
